# Effects of pregabalin added to a multimodal anesthetic protocol in cats undergoing ovariohysterectomy

**DOI:** 10.3389/fvets.2026.1910676

**Published:** 2026-09-30

**Authors:** Margherita Romano, Ismael Hernández-Avalos, Celeste Nanci, Stella Costa, Antonio Lombardo, Simona Di Pietro, Filippo Spadola, Claudia Interlandi

**Affiliations:** 1 Veterinary Clinic "City of Catania", Vetpartners, Catania, Italy; 2 Clinical Pharmacology and Veterinary Anesthesia, Biological Sciences Department, Facultad de Estudios Superiores Cuautitlán, Universidad Nacional Autónoma de México (UNAM), Cuautitlán, Mexico; 3 Department of Veterinary Sciences, University of Messina, Messina, Italy

**Keywords:** anxiety, cats, pregabalin, recovery, sedation, stress

## Abstract

Advancement in anesthetic protocols is necessary to address the evolving challenges of perioperative cats’ management in veterinary practice. In this study, 54 cats were enrolled and were randomly assigned to either Group P (Pregabalin, PGB) or Group C (Control). Group P received 5 mg/kg of PGB orally 90 min before hospital admission, while Group C received identical empty capsules. Sedation was assessed using a Feline Multiparametric Sedation Scale and a Simple Qualitative Scale. All cats were premedicated with methadone (0.2 mg/kg IM) and alfaxalone (1 mg/kg IM). Anesthesia was induced with propofol at 2 mg/kg and maintained with isoflurane. Physiological parameters (HR, RR, SpO2, EtCO2, Blood Pressure, and Temperature) were monitored throughout surgery. In the immediate postoperative period, the Glasgow Feline Composite Measure Pain Scale short-form and the Feline Grimace Scale were used, measured at 0, 15, 30, and 60 min after extubation. Statistical analysis included Two-way ANOVA for physiological data and Wilcoxon-Mann–Whitney tests for pain and sedation scores. Sedation scores increased in Group P by 2.46 (95% CI: 1.69 to 3.23; *p* < 0.001). Conversely, the scores of the scales used in the immediate postoperative period were significantly lower in the Group P at all evaluation times −2.15 (95% CI: −2.92 to −1.39; *p* < 0.001). No significant differences were observed in physiological parameters. In conclusion, prehospital administration of PGB provides effective sedation and improves postoperative comfort, making it a valuable adjunct to feline anesthesia protocols.

## 1 Introduction

Because its benefits and potential complications are well documented, elective ovariohysterectomy (OHE), a standardized surgical procedure frequently performed in general veterinary practice, is widely used as a clinical model for evaluating acute pain (1–3). The concept of preventive analgesia, aimed at reducing the intensity and duration of postoperative pain, has been demonstrated by several authors, who have provided evidence supporting the existence of a central component of hypersensitivity to pain following injury (4, 5). Various antinociceptive techniques applied before injury have proven more effective in reducing post-injury central sensitization than those administered after injury (6). Domestic cats are often exposed to stressful stimuli and resist veterinary handling, showing signs of discomfort during both the preoperative and immediate postoperative period (7). Pregabalin (PGB) and gabapentin (GBP) are anticonvulsant drugs with proven clinical efficacy in humans and for alleviation of acute anxiety and fear related to transportation and veterinary visits in cats (8). Both agents are structural analogues of gamma-aminobutyric acid (GABA) and bind to the alpha 2-delta (α2-δ) subunit of voltage-dependent calcium channels. Compared to GBP, which has been more widely used in veterinary medicine, PGB exhibits a greater affinity for the α2-δ protein subunit of voltage-gated calcium channels (9). While the pharmacokinetics of PGB have been characterized in humans (10–13), dogs (14), and cats (8, 15), evidence regarding its postoperative efficacy remains limited due to a lack of robust clinical data (16).

The perioperative management of cats is often compromised by stress (17, 18). Therefore, administration of additional oral premedication prior to general anesthesia and surgery may be considered to facilitate cats’ management. This has been shown to reduce fear and anxiety, improving the animal’s experience without significantly affecting hemodynamic or cardiographic variables (10, 19). This study aimed to evaluate the effects of a single oral dose of PGB as an adjunctive premedication in cats undergoing OHE, and to determine its influence on sedation, physiological variables, and postoperative pain scales during the first hour of recovery. The hypothesis was that PGB, in addition to its sedative effect, could influence postoperative assessment scores, thereby improving the quality of recovery during the initial phase of hospitalization.

## 2 Materials and methods

### 2.1 Animals

This prospective, randomized, double-blind, placebo-controlled clinical trial was reviewed and approved in accordance with the standards recommended by the Guide for the Care and Use of Laboratory Animals and Directive 2010/63/EU for animal experiments (approval number: 074/2022), and informed consent was obtained from the owners prior to the enrollment of all cats. Fifty-four (*n* = 54) domestic shorthair cats, with a mean body weight of 3.5 ± 1.3 kg and a mean age of 12.2 ± 13 months, were enrolled in the study. These cats were presented at the Veterinary Clinic for elective OHE between April and October 2024. Animals were deemed healthy based on physical examinations and laboratory analyses performed a few days before surgery. Inclusion criteria were a bodyweight > 1 kg and an American Society of Anesthesiologists (ASA) physical status of I. Cats were excluded if they presented with pre-existing painful conditions, systemic diseases (ASA III or higher), or a history of organ failure. Additionally, subjects undergoing pharmacological treatment within the previous 14 days, those requiring laparoscopic sterilization, or those with behavioral issues hindering clinical assessment were excluded. Food was withheld for 4–6 h prior to surgery, while water was provided ad libitum. On the day of the procedure, the animals were admitted.

### 2.2 Study design and treatments

Before the study began, an independent researcher not involved in the recruitment or evaluation of the subjects used online software (www.randomization.com) to generate the randomization sequence, assigning cats to either the control group (Group C) or the PGB group (Group P). For Group P, pregabalin (PGB; Lyrica 25 mg; Pfizer) was administered at a dose of 5 mg/kg using capsules customized according to body weight and prepared with an analytical balance (Balance XPR204; Mettler-Toledo) (11, 19). PGB was administered at home by the owners approximately 60 minutes before hospital arrival and subsequent premedication/induction of anesthesia. This timing was intentionally chosen to coincide with the peak pharmacokinetic plasma concentrations (TMax of 0.5–1 h) described by Lamminen et al. (10), ensuring that the maximal sedative and anxiolytic effects of the drug were already established during transport and at the beginning of the monitoring period (17, 19). Group C received empty capsules (placebo) identically. Capsule administration was followed by 2 mL of water via syringe to facilitate swallowing. Complete ingestion of the capsule was confirmed by each owner.

### 2.3 Anesthesia

Cats included in this study were premedicated with methadone 0.2 mg/kg (Comfortan 10 mg/mL, Dechra Veterinary Products, UK) followed by alfaxalone 1 mg/kg (Alfaxan 10 mg/mL, Jurox Zoetis, Belgium S. A.), both administered intramuscularly (IM). After pre-medication, the cats were kept in a quiet environment for approximately 15 min, during which time the degree of sedation achieved was assessed. If sedation was deemed inadequate according to the Feline Multiparametric Sedation Scale (FMSS) and Simple Qualitative Scale (SQS) assessment scales, a supplemental IM dose of dexmedetomidine 5 μg/kg (Dexdomitor 0.5 mg/mL, Zoetis Orion Pharma, Italy) was administered, as previously described in similar multimodal protocols (17, 20).

Once adequate sedation was achieved, a 24-gauge intravenous (IV) catheter (0.74 mm; Farmacare SrL, San Pietro in Casale, Italy) was aseptically placed in the cephalic vein and secured for drug administration. Lactated Ringer’s crystalloid solution (Baxter’s lactated Ringer’s solution) was administered intravenously at a constant rate of 3 mL/kg/h throughout the surgical procedure. Anesthesia was induced with propofol (Proposure 10 mg/mL; Boehringer Ingelheim Animal Health, Italy) administered intravenously (IV) at a dose of 2 mg/kg over approximately 60 s. Following orotracheal intubation with an appropriately sized endotracheal tube (ETT), cats were transferred to the operating room and connected to a non-rebreathing circuit for maintenance of anesthesia. Anesthesia maintenance was achieved through the vaporization of isoflurane (Isoflo; Zoetis, Italy) delivered in 100% oxygen, with the dial set to 2.3 ± 0.13%, adjusting this concentration according to the required anesthetic depth to maintain a mean arterial pressure (MAP) of 60–90 mmHg. The vaporizer was adjusted to maintain a surgical plane of anesthesia, which was monitored by assessing clinical signs, including jaw tone, palpebral reflex, eyeball position, limb withdrawal reflex, and the absence of spontaneous movement. Remifentanil hydrochloride (Ultiva 1 mg; GlaxoSmithKline Manufacturing S.p.A., Italy) was available for intraoperative rescue analgesia if required, administered as a 1 μg/kg bolus, followed by a maintenance CRI of 0.1–0.5 μg/kg/min (21–23).

### 2.4 Sedation score

After anesthetic premedication, all cats were assessed by three observers who were blinded to the sedation protocol. Sedation scores were evaluated using a previously validated feline multiparametric sedation scale (FMSS), which assessed posture, behavior, response to sound, and response to restraint for intramuscular (IM) injection or intravenous (IV) catheter placement (6, 24, 25). Each observer used the cumulative score to classify sedation levels as follows: 0–3 (absent), 4–6 (mild), 7–9 (moderate), and 10–12 (profound). The final score ranged from 0 to 12, with 0 representing no sedation and 12 representing deep sedation (17, 25, 26). Furthermore, a simple qualitative scale (SQS) was used to define the sedation level as absent, mild, moderate, or profound (25). The sequence of procedures for assessing sedation was consistent across all cats.

### 2.5 Physiological parameters

Physiological parameters were recorded after premedication (Tp), after induction (Ti), and at 5-min intervals from the start of anesthesia until cats extubation (T5, T10, T15, and T20). Heart rate (HR) was assessed via ECG monitoring and expressed as beats per minute (bpm). Non-invasive blood pressure (systolic, diastolic, and mean; SAP, DAP, and MAP, respectively; mmHg) was measured oscillometrically (Suntech® Vet20, USA) using a neonatal cuff with a width equal to 40% of the tail circumference. Peripheral oxygen hemoglobin saturation (SpO2; %) was assessed using a pulse oximeter probe attached to the tongue. Respiratory rate (RR; breaths per minute), end-tidal CO₂ (EtCO₂; mmHg) and end-tidal fraction of isofluorane (ETIso) were measured using a side-stream capnograph and displayed on the anesthesia machine (GE Da-tex-Ohmeda Avance 6.8 with Datex-Ohmeda S/5; M-CAiOV; Helsinki, Finland). Body temperature (T, °C) was monitored using a calibrated thermistor inserted into the oesophagus and was maintained between 38.5 °C and 37.0 °C with a circulating warm water blanket (Soarmed Medical-Tech Co., Ltd., Taiwan). Standard methodology was used for preoperative preparation and surgical procedures, all of which were performed by the same surgical team. At the end of surgery, all cats received meloxicam 0.1 mg/kg subcutaneously (Metacam 0.5/ml, Boehringer Ingelheim Animal Health Italia S.p. A.), followed by 0.05 mg/kg orally every 24 h for 4 days.

### 2.6 Postoperative pain assessment

Pain assessments were conducted during the immediate postoperative period at four time points: immediately after extubation (T0), followed by evaluations at 15, 30, and 60 min (T15, T30, and T60, respectively). The assessments were performed by three observers who were blinded to the treatment groups. Two validated pain scoring systems were used to assess postoperative pain. All cats were assessed using a shortened version of the Glasgow Feline Composite Measure Pain Scale (CMPS-Feline) and the Feline Grimace Scale (FGS). The CMPS-Feline is a psychometrically validated clinical tool designed to provide objective, repeatable assessments of acute postoperative pain in cats.

The scale evaluates six behavioral categories, assigning each a numerical score based on the intensity of the observed signs. The maximum score is 20; a score ≥5/20 was established as the cut-off point for administering rescue analgesia (6, 27–30). The FGS is a non-invasive instrument used to assess facial expressions for determining the need for analgesic intervention. A total score of ≥4/10 was established as the intervention threshold (6, 31). Cats who reached or exceeded the intervention threshold on the CMPS-Feline or FGS scales would be administered rescue analgesia (buprenorphine 0.02 mg/kg IV; Bupaq 0.3 mg/mL, Virbac, Richter Pharma, Italy). Throughout the study period, cats were monitored for adverse effects (ataxia, vomiting, diarrhea) (32).

### 2.7 Statistical analysis

A power analysis was performed to determine the number of cats required for this study. Sample size was calculated using G*Power 3.1 software (Heinrich-Heine-Universität Düsseldorf, Germany). To detect a clinically significant difference of 2 points on the Glasgow Composite Measure Pain Scale-Feline (CMPS-Feline), assuming a standard deviation of 2.5 based on a preliminary study corresponding to an effect size (Cohen’s d) of 0.8 with a power (1-β) of 0.80 and a significance level (α) of 0.05, using the Wilcoxon-Mann–Whitney test (two groups), a minimum of 26 cats per group was required. A large effect was assumed as reported in a previous publication (33). To account for a potential 15% attrition (drop-out) rate, the final recruitment target was set at 27 cats per group (total *n* = 54). Statistical analyses were performed using commercially available software (GraphPad Prism version 8.2.1; GraphPad Software Inc., La Jolla, CA, USA, and SPSS version 26.0; SPSS Inc., Chicago, IL, USA). Data were analyzed for normality using the Shapiro–Wilk test and reported as mean ± SD or median (range), as appropriate. Clinical parameters (HR, SpO₂, SAP, MAP, DAP, RR, EtCO2, and T) were analyzed using a two-way mixed ANOVA with repeated measures (time as the within-subject factor and group as the between-subject factor). When significant differences were detected, Bonferroni’s post-hoc pairwise comparison test was used. Pain scores (FMSS, CMPS-Feline, and FGS) were reported as Median (IQR). Differences over time within each group were analyzed using the Friedman test followed by Dunn’s post-hoc test. Comparisons between the two groups at each time point were performed using the Wilcoxon-Mann–Whitney test and the effect size was reported as the rank-biserial correlation (r_rd_). For the primary comparisons between groups, the mean difference and its 95% confidence interval (95% CI) were calculated to provide an estimate of the effect size and precision of the results. Statistical significance was set at *p* < 0.05. The two scales used to assess postoperative pain were compared using Spearman’s rank correlation coefficient to evaluate the strength of their association. Kendall’s coefficient of concordance W was calculated to measure the degree of agreement among observers. At each measured time point, the scores of the three observers were averaged.

## 3 Results

Data from all cats enrolled in the study were analyzed (Figure 1). A total of 54 (*n* = 54) female cats undergoing elective OHE were enrolled in the study over 7 months. There were no significant differences between groups regarding age, body weight, anesthesia time, and duration of surgery (Table 1). The cats completed the study without complications. Furthermore, no signs of transient ataxia or motor disturbances were observed in cats, and no incoordination or gait abnormalities were reported by owners during the 24-h follow-up period. Statistically significant differences in sedation scores were observed between the two groups. Specifically, when assessed using the FMSS, scores were significantly lower 2.46 (95% CI: 1.69 to 3.23; *p* < 0.001) in Group C (median: 8; IQR: (6–10) r_rd_ = − 0.76) compared to Group P (median: 11; IQR: (8–12)) (see Figure 2). The same results were observed using the SQS, in which “moderate sedation” responses prevailed in Group C (87%), compared to “profound sedation” responses in Group P (93%) (Table 2, Figure 3). None of the cats required preoperative dexmedetomidine, as all cats achieved adequate sedation before induction of general anesthesia. Monitoring of physiological parameters during anesthesia showed no significant differences between the two groups regarding HR, SpO_2_, RR, EtCO₂, SAP, MAP, DAP, ETIso and T° (Table 3). In Group C, HR decreased from a baseline Tp of 175 ± 14 beats/min to 144 ± 14 beats/min at T20 (with intermediate values of 158 ± 19 at Ti, 148 ± 16 at T5, 146 ± 14 at T10, and 145 ± 16 at T15), a comparable physiological decrease was observed in Group P, shifting from 168 ± 13 to 138 ± 17 beats/min. Regarding SAP, values in Group C went from 130 ± 14 mmHg at Tp to 121 ± 11 mmHg at T20, while in Group P it went from 122 ± 12 mmHg at Tp to 111 ± 13 mmHg at T20. MAP ranged from 102 ± 9 mmHg at Tp down to 80 ± 10 mmHg at T20 in Group C, and from 96 ± 12 mmHg at Tp to 82 ± 11 mmHg at T20 in Group P. DAP values progressed from 79 ± 11 to 66 ± 7 mmHg in Group C, and from 72 ± 14 to 65 ± 6 mmHg in Group P from Tp to T20. RR remained stable throughout, moving from 24 ± 6 to 21 ± 4 breaths/min in Group C, and from 21 ± 3 to 20 ± 4 breaths/min in Group P, ETCO2, SpO2, and ETIso showed comparable trends between groups. Specifically, ETIso decreased progressively from induction 2.03 ± 0.08% vs. 2.04 ± 0.04%) to the end of monitoring (1.67 ± 0.07% vs. 1.63 ± 0.08%) in Groups C and P, respectively. T° decreased in all cats by 1.7 ± 0.8 degrees (mean ± SD). Overall, these parameters showed a constant trend and remained within the normal clinical range throughout the monitoring period in both groups. Mean pain scores during the immediate postoperative period, as assessed by the CMPS-Feline and FGS, were significantly higher in Group C compared to Group P. Indeed, a statistically significant difference was found between Group C and Group P at all monitoring time points (*p* < 0.001). Group C also showed significant intragroup differences throughout the monitoring period. The score in all groups did not exceed the cutoff point value and did not require rescue analgesia. Regarding the CMPS-Feline, Group C showed a significant increase in scores compared to the beginning of monitoring (T0) for items: 4, 4b (*p* < 0.05) at T15, and 2, 4, 4b (*p* < 0.001) and 3 (*p* < 0.05) at T30, and 2, 4, 4b (*p* < 0.001), and 3 (*p* < 0.05) at T60, (Table 4, Figure 2). A significant difference in pain scores was observed between groups (mean difference: -2.15; 95% CI: −2.928 to −1.390; *p* < 0.001), indicating a clinically relevant reduction in the treatment group. Similarly, FGS scores in Group C showed significant differences across all time points (T15, *p* < 0.05; T30 and T60, *p* < 0.001) and significant difference in pain scores was observed between groups – 1.76 (95% CI: −2.80 to −0.72; *p* = 0.006). No significant changes were observed within Group P (Table 5, Figure 2). None of the cats required postoperative rescue analgesia with buprenorphine. Spearman’s rank correlation indicated a weak association between the CMPS-Feline and FGS scores. The correlation was weak at T0 (*r* = 0.2), at T15 (*r* = 0.08), at 30 min (*r* = 0.4), and 60 min (*r* = 0.2). An excellent degree of concordance was found among observers (Kendall’s *W* = 0.8, *p* < 0.001).

**Figure 1:**
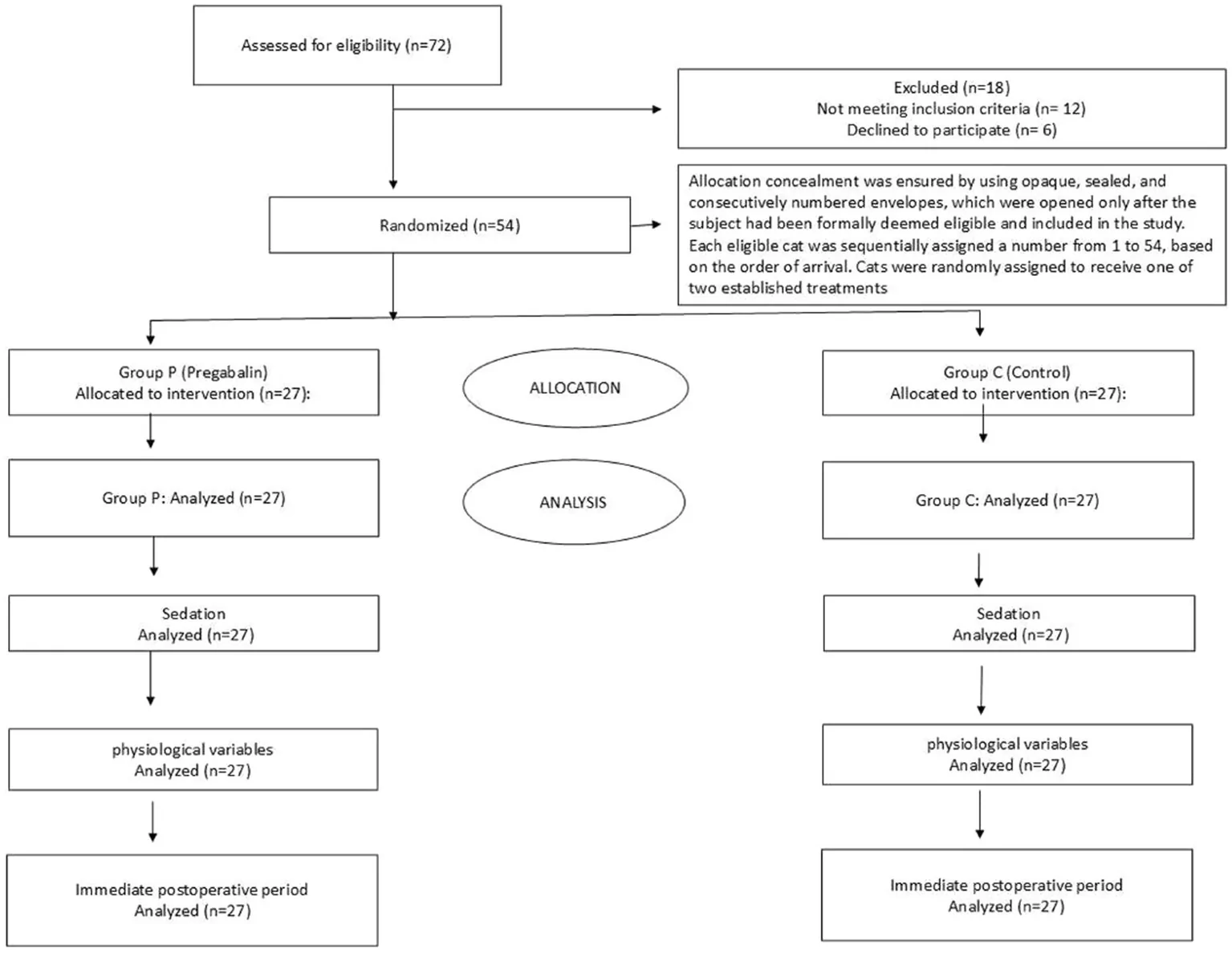
CONSORT flow diagram. Flow diagram of the progress through the phases of the study.

**Table 1:** Demographic data of 54 cats that underwent anesthesia and an ovariohysterectomy.

		Groups (*n* = 54)		
Demographic	Pregabalin (*n* = 27)		Placebo (*n* = 27)	*p-*value
Age (m)	9.9 ± 2.8		14 ± 17	0.9
Weight (kg)	3.5 ± 0.4		3.4 ± 0.6	0.7
Anesthesia time (minutes)	35.5 ± 3.4		35.1 ± 2.7	0.8
Surgery time (minutes)	17 ± 2.1		18 ± 2.6	0.5

Each cat was assigned to receive either PGB or a placebo, administered PO before premedication. Data are presented as mean ± SD. Anesthesia time was recorded as the time from premedication to reversal.

**Figure 2:**
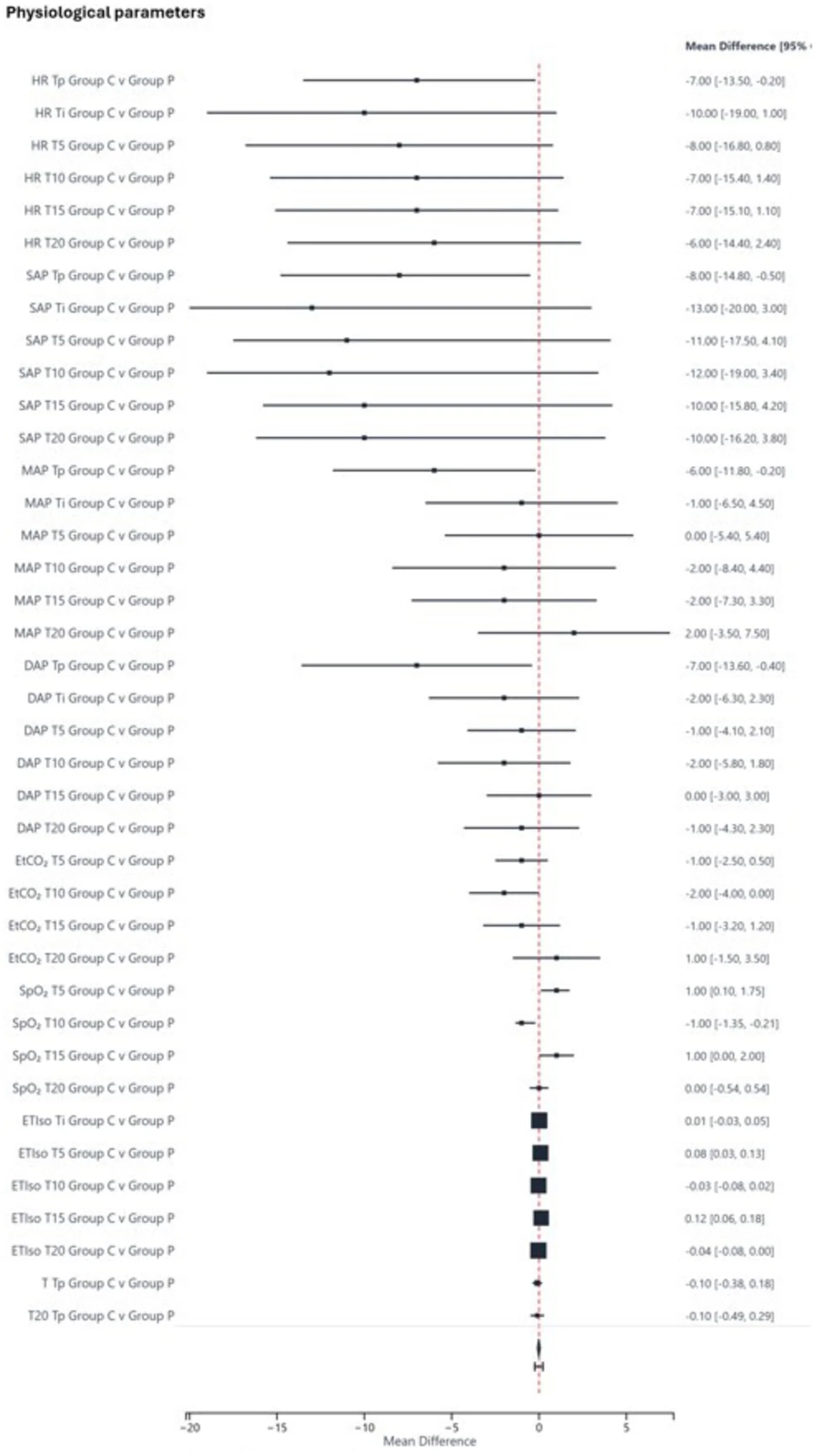
Interval plot of mean differences (Group P vs. Group C) with 95% confidence intervals for physiological parameters across different time points.

**Table 2:** Feline Multiparametric Sedation Scale (FMSS) and Simple Qualitative Scale (SQS) scores assessed after anesthetic premedication in female cats undergoing ovariohysterectomy.

Sedation score	Group P	Group C	*p-*value, rrb
FMSS	11 (8/12)	8 (6/10)	<0.0001, rrb = − 0.76

SQS	Moderate sedation	Profound sedation	Mild	Moderate	Profound
	(7%)	(93%)	Sedation (10%)	Sedation (87%)	Sedation (3%)

Data are expressed as median (IQR) for FMSS and as percentages for SQS in Group P and Group C. Comparisons between groups were performed using the Mann–Whitney U test. *p*-values < 0.05 were considered statistically significant, rrb represents the rank-biserial correlation coefficient (effect size).

**Figure 3:**
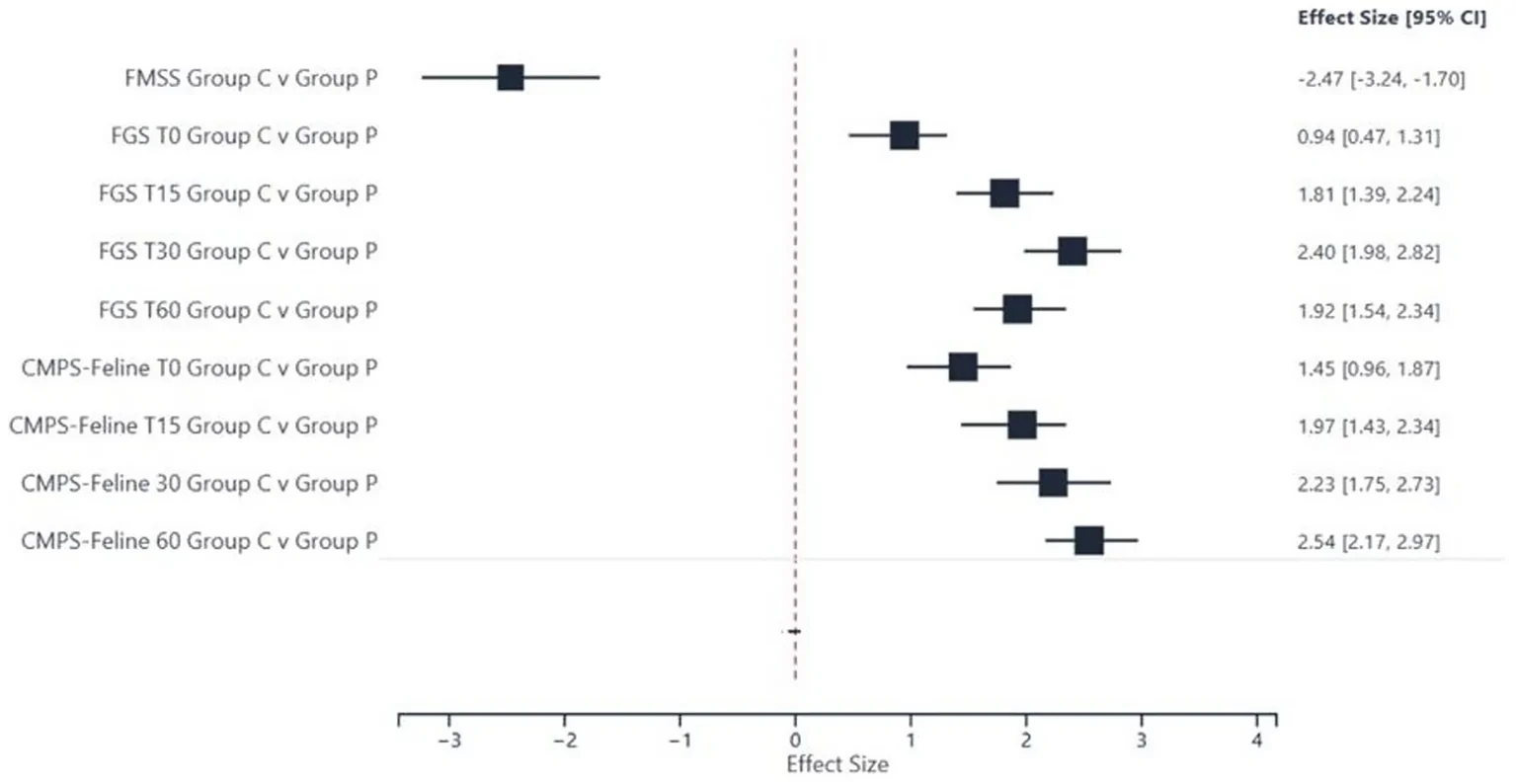
Interval plot of effect sizes (with 95% confidence intervals) across the evaluation scales (FMSS, CMPS-Feline, and FGS) at different time points.

**Table 3:** Physiological variables expressed as mean ± SD for HR, RR, SAP, MAP, DAP, EtCO2, SpO2, ETIso, and T° in Group C and Group P.

Variables	Groups	Tp	Ti	T5	T10	T15	T20
HR (Beats/Minute)	C	175 ± 14	158 ± 19	148 ± 16	146 ± 14	145 ± 16	144 ± 14
	P	168 ± 13	148 ± 18	140 ± 17	139 ± 18	138 ± 16	138 ± 17
SAP (mmHg)	C	130 ± 14	127 ± 15	123 ± 12	124 ± 15	123 ± 11	121 ± 11
	P	122 ± 12	114 ± 13	112 ± 13	112 ± 15	113 ± 11	111 ± 13
(mmHg)	C	102 ± 9	85 ± 11	81 ± 10	81 ± 13	82 ± 10	80 ± 10
	P	96 ± 12	84 ± 10	81 ± 11	79 ± 9	80 ± 10	82 ± 11
DAP (mmHg)	C	79 ± 11	68 ± 9	65 ± 6	67 ± 8	66 ± 6	66 ± 7
	P	72 ± 14	66 ± 7	64 ± 6	65 ± 7	66 ± 6	65 ± 6
RR (Breaths/Minute)	C	24 ± 6	20 ± 4	21 ± 2	20 ± 2	19 ± 3	21 ± 4
	P	21 ± 3	21 ± 5	20 ± 2	19 ± 3	19 ± 5	20 ± 4
ETCO2 (mmHg)	C			33 ± 2	34 ± 3	35 ± 4	33 ± 3
	P			32 ± 3	32 ± 4	34 ± 4	34 ± 5
SPO2 (%)	C			98 ± 1	99 ± 1	98 ± 2	99 ± 1
	P			99 ± 1	98 ± 1	99 ± 1	99 ± 1
ETISO (%)	C		2.03 ± 0.08	1.85 ± 0.09	1.81 ± 0.09	1.62 ± 0.13	1.67 ± 0.07
	P		2.04 ± 0.04	1.93 ± 0.09	1.78 ± 0.09	1.74 ± 0.08	1.63 ± 0,08
T° (Body Temperature)	C	38.6 ± 0.4					37.6 ± 0.7
	P	38.5 ± 0 6					37.5 ± 0.8

Physiological parameters were recorded after premedication (Tp), after induction (Ti), and at 5-min intervals from the start of anesthesia until patient extubation (T5, T10, T15, and T20).

**Table 4:** Glasgow Feline Composite Measure Pain Scale (CMPS-Feline): the scale evaluates the following behavioural categories, assigning a numerical score to each based on the intensity of the observed sign: vocalisation; posture; attention to the surgical site; activity and movement; response to palpation; mood and mental status.

Items	T0		T1		T 3		T 60	
	Group C	Group P	Group C	Group P	Group C	Group P	Group C	Group P
1	0 (0/1)	0 (0/0)	0 (0/1)	0 (0/0)	0 (0/1)	0 (0/0)	0 (0/1)	0 (0/0)
2	0 (0/2)	0 (0/1)	0 (0/2)	0 (0/1)	0 (0/2)	0 (0/1)*	0 (0/2)	0 (0/1)*
3	0 (0/1)	0 (0/0)	0 (0/1)	0 (0/0)	0 (0/2)	0 (0/0)*	0 (0/2)	0 (0/0)*
4	0 (0/1)	0 (0/1)	0 (0/2)	0 (0/1)*	0 (0/2)	0 (0/1)*	0 (0/2)	0 (0/1)*
4bis	0 (0/1)	0 (0/1)	0 (0/2)	0 (0/1)*	0 (0/2)	0 (0/1)*	0 (0/2)	0 (0/1)*
5	0 (0/2)	0 (0/1)	0 (0/2)	0 (0/1)	0 (0/2)	0 (0/1)	0 (0/2)	0 (0/1)
6	0 (0/1)	0 (0/0)	0 (0/1)	0 (0/0)	0 (0/1)	0 (0/0)	0 (0/1)	0 (0/0)
7	0 (0/1)	0 (0/0)	0 (0/1)	0 (0/0)	0 (0/1)	0 (0/0)	0 (0/1)	0 (0/0)
Statistical comparison (*p*, rrb)	*p* < 0.0001, rrb = 0.78		*p* < 0.0001, rrb = 0.95		*p* < 0.0001, rrb = 0.9		*p* < 0.0001, rrb = 1	

The scores obtained in the two groups with the individual items at 0, 15, 30, and 60 min are expressed as Median (IQR). *Significant differences between the groups (*p* < 0.05), r_rd_ represents the rank-biserial correlation coefficient (effect size).

**Table 5:** Feline grimace scale (FGS).

Time (minutes)	Group P	Score	Group C	*p*-value, rrb
T0	0 (0/1)		2 (0/3)*	< 0.001, 0.86
T15	0 (0/1)		2 (0/3)*^, ♣^	< 0.001, 0.53
T30	0 (0/1)		3 (1/3)*^, ♣^	< 0.001, 1
T60	0 (0/2)		3 (2/3)*^, ♣^	< 0.001, 0.94

The total scores obtained in the two groups at T0, T15, T30, and T60 minutes are expressed as Median (IQR). * Comparisons between groups were performed using the Mann–Whitney *U*-test, *p*-values < 0.05 were considered statistically significant, r_rd_ represents the rank-biserial correlation coefficient (effect size).

^♣^Significant differences within groups (T15, *p* < 0.05; T30 and T60, *p* < 0.001).

## 4 Discussion

This study evaluated the effects of preoperative PGB administration in cats undergoing OHE. The results demonstrated that the adjunctive administration of a single 5mg/kg oral dose of PGB contributed to profound sedation, suggesting enhanced cat management when combined with the pre-anesthetic protocol. Furthermore, cats in the Group P showed lower CMPS-Feline and FGS scores during the first hour of postoperative monitoring; however, this effect should be considered in the context of a multimodal protocol that included pre-anesthetic methadone and postoperative meloxicam. This finding is clinically relevant because the immediate recovery phase represents a critical transition window for cats; a phase during which most anesthesia-related complications occur. The lower pain scores on the aforementioned scales, observed in the Group P, likely contributed to improved perioperative management during this vulnerable period (34, 35). These findings align with Lamminen et al. (10), who demonstrated that 5 mg/kg of PGB effectively alleviates acute anxiety and fear in cats during transport and veterinary visits. By administering the drug in a familiar environment prior to travel, as suggested by several authors, the cats arrived at the facility with pharmacological effects already manifest, thereby facilitating initial handling and mitigating transport-induced stress (19, 36, 37). These observations are further supported by Miranda-Cortés et al. (7), Li et al. (11), and Esteban et al. (15), who reported that a single oral dose of PGB induces mild to moderate sedation, an effect clearly reflected in the sedation scores employed in this protocol. This further clarifies the significant differences in sedation levels observed between the Group P and Group C (11). Although the sedative effect of pregabalin is documented in the literature (10, 19), in order to preserve observer blinding and prevent potential measurement bias, the degree of sedation was indirectly assessed through the standardized response to the co- administration of methadone and alfaxalone. This methodological approach allowed for monitoring the synergistic effect of the drugs on the overall sedation level while keeping the identity of the study treatment concealed from the clinical evaluators. The clinical efficacy observed in this study is supported by the favorable pharmacokinetic profile of PGB in felines (14, 15). PGB administration did not significantly alter the physiological parameters monitored during anesthesia (HR, RR, SAP, MAP, and DAP), all of which remained within the reference range for anesthetized cats (38, 39). These findings, along with previous reports on gabapentinoids, confirm that the 5 mg/kg protocol used in this study is cardiovascularly safe and does not interfere with the cat’s hemodynamic equilibrium during surgical procedures such as OHE (40). Regarding hemodynamic safety, the findings showed a slight decrease in blood pressure in Group P, which remained within the physiological range for anesthetized cats, with no statistically significant differences between the two groups. It is worth noting that while Li et al. (11) observed hypotension in healthy cats administered PGB at 5 and 10 mg/kg, the cats in this study remained clinically stable. This difference might be attributed to the specific perioperative context and the multimodal anesthetic protocol employed in this study, which may have mitigated the significant hypotensive effects reported in non-surgical settings. These observations, combined with the lack of significant changes in echocardiographic variables reported by Li et al. (11), suggest that while blood pressure should be carefully monitored, PGB remains a safe option for feline sedation and analgesia. The hemodynamic stability observed in this study is consistent with the broader literature on gabapentinoids in felines. As highlighted in a recent systematic review by Laguardia et al. (39), gabapentinoids are characterized by a favorable safety profile, with minimal impact on cardiovascular parameters, even when used for sedation or analgesia. Although the comparison of ETiso requirements showed no significant differences between the two groups, these results should not be interpreted as definitively ruling out an isoflurane-sparing effect. To date, there is limited information in the literature on the impact of PGB on anesthetic requirements in cats; therefore, further studies are needed to better characterize its potential role in reducing the need for inhalant anesthetics (41). While GBP has been the primary focus of such research, the greater potency and more predictable pharmacokinetic profile of PGB, which requires significantly lower dosages to achieve comparable clinical effects, represent a significant advancement in feline perioperative care (39, 42). Effective pain management is essential to prevent unnecessary suffering and physiological stress (43); indeed, several studies have shown that unmanaged pain can worsen the incidence of complications in the immediate postoperative period (34, 35, 44). OHE is known to cause significant postoperative pain; therefore, this study evaluated the clinical efficacy of PGB when added as a single oral dose to a multimodal protocol to optimize the analgesic profile during the early recovery phase. The mean difference in pain scores was −2.15 (95% CI: −2.928 to −1.390; *p* < 0.001). Since the entire confidence interval remains well below zero, these results not only confirm statistical significance but also demonstrate a reduction in pain scores in Group P, reflecting a significant improvement in cats’ comfort during the recovery phase.

The clinical relevance of these findings must also take into account the importance of observer assessments of treatment efficacy. Indeed, differences between observers in pain assessments may lead to different conclusions regarding treatment efficacy (45, 46). The observers had previous experience assessing pain and sedation in cats, so the high concordance found (Kendall’s *W* = 0.8, *p* < 0.001) suggests that all observers observed better recovery in group P cats.

Although both scales assigned the lowest pain scores to Group P, it should be considered that it is not clear whether these results were due to the preventive analgesic efficacy of the protocol used or whether they were also influenced by the residual pharmacological effects of PGB administration (31, 32, 35).

While the use of PGB for postoperative pain management in cats is still a developing topic, its potential role in mitigating central sensitization and the wind-up phenomenon is of clinical interest (4–6, 18, 47–50). However, these findings remain preliminary, as they are limited to the clinical assessment of the immediate postoperative period (the first hour after extubation) and focus on clinical outcomes rather than mechanistic pathways.

Although pain scores in both groups remained below the intervention threshold for rescue analgesia, the PGB group showed lower scores during the first postoperative hour. From a clinical perspective, this suggests that adding PGB could optimise a cat's comfort during the critical transition from anesthesia to consciousness. As in previous studies, minimizing even subthreshold pain is essential in feline medicine to reduce the cumulative stress response and promote a smoother recovery. The possibility that a single preoperative dose of PGB improves recovery, even as part of a multimodal approach, underscores its value in the perioperative management of cats (34, 35, 47, 51). The results obtained with the Spearman correlation highlighted a weak correlation between the Glasgow CMPS-Feline and the Feline Grimace Scale (FGS). Although both scales correctly identified Group C as having higher scores, the numerical correlation between them remained low throughout the monitoring period. This discrepancy may be attributed to the different aspects assessed by each tool, confirming that they should be used complementarily rather than interchangeably to ensure a comprehensive assessment of the feline (27, 29, 31, 46, 51). This study is subject to several limitations. First, plasma PGB concentrations were not measured, which limited the interpretation of its pharmacokinetics. Second, the postoperative observation period was short; although 24-hour monitoring would have provided valuable information on the duration of pregabalin’s analgesic effects, the owners' request for early discharge once their cats were deemed clinically stable prevented a standardized long-term evaluation for the entire group.

Therefore, extending the follow-up period in future research is warranted to fully characterize the drug’s long-term efficacy and safety profile. Regarding pain assessment, a further limitation is the lack of objective monitoring of nociception, such as the parasympathetic tone activity (PTA) index. The study was based on behavioral pain scales, and to date, it is still unclear how different sedation protocols may influence postoperative pain scores when using various subjective scales (31). Furthermore, the single-center design and the inclusion of a restricted population specifically young, healthy cats undergoing only ovariohysterectomy, with the exclusion of cats with chronic pain and those with an ASA physical status higher than I may limit the external validity and generalizability of the results to more complex or diverse clinical populations. Finally, in the present clinical study, the evaluation of the effects of anesthesia and surgery separately on pain scores was not possible for ethical reasons. The inclusion of sham and negative control groups undergoing general anesthesia without the surgical procedure, or surgery without the administration of analgesics, was not a possibility. Thus, the methodology of the study is limited by its nature as a clinical study involving surgery, which necessitates the administration of anesthetics.

## 5 Conclusion

In conclusion, adding a single oral dose of PGB to a multimodal protocol promoted effective sedation and a stable trend in physiological variables during anaesthesia, which remained within normal clinical limits. It also improved the quality of early recovery. Pregabalin may be a useful adjunct in multimodal perioperative pain management in healthy cats undergoing OHE.

## Data Availability

The original contributions presented in the study are included in the article/supplementary material, further inquiries can be directed to the corresponding author.

## Glossary

PGB: Pregabalin
GBP: Gabapentin
GABA: Gamma-Aminobutyric Acid
ASA: American Society of Anesthesiologists
IM: Intramuscular
IV: Intravenously
FMSS: Feline Multiparametric Sedation Scale
SQS: Simple Qualitative Scale
ETT: Endotracheal Tube
RR: Respiratory rate
SAP, MAP, DAP: Systolic, Diastolic and Mean arterial pressure
SpO2: Haemoglobin oxygen saturation
HR: Heart rate
T: Temperature
EtCO2: End-tidal CO2
EtIso: End-tidal fraction of isoflurane
CMPS-Feline: Glasgow Feline Composite Measure Pain Scale
SD: Standard deviation
FGS: Feline Grimace Scale
IQR: Interquartile Range
CMax: Mean Maximum plasma concentration
TMax: Time to Maximum Concentration
CNS: Central Nervous System
NMDA: N-methyl-D-aspartate
TRPV: Transient Receptor Potential
